# Endovascular ischemic stroke models of adult rhesus monkeys: a comparison of two endovascular methods

**DOI:** 10.1038/srep31608

**Published:** 2016-08-18

**Authors:** Di Wu, Jian Chen, Bincheng Wang, Mo Zhang, Jingfei Shi, Yanhui Ma, Zixin Zhu, Feng Yan, Xiaoduo He, Shengli Li, David Dornbos III, Yuchuan Ding, Xunming Ji

**Affiliations:** 1 China-America Institute of Neuroscience, Xuanwu Hospital, Capital Medical University, Beijing, 100053, China; 2 Center of Stroke, Beijing Institute for Brain Disorders, Beijing, China; 3 Department of Neurosurgery, Xuanwu Hospital, Capital Medical University, Beijing, China; 4 Department of Radiology, Xuanwu Hospital, Capital Medical University, Beijing, 100053, China; 5 Department of Anesthesiology, XuanWu Hospital, Capital Medical University, Beijing 100053, China; 6 Department of Laboratory Animal Science, Capital Medical University, Beijing, China; 7 Department of Neurological Surgery, the Ohio State University Wexner Medical Center, Columbus, OH, USA; 8 Department of Neurological Surgery, Wayne State University School of Medicine, Detroit, MI, USA

## Abstract

To further investigate and improve upon current stroke models in nonhuman primates, infarct size, neurologic function and survival were evaluated in two endovascular ischemic models in sixteen rhesus monkeys. The first method utilized a micro-catheter or an inflatable balloon to occlude the M1 segment in six monkeys. In the second model, an autologous clot was injected via a micro-catheter into the M1 segment in ten monkeys. MRI scanning was performed on all monkeys both at baseline and 3 hours after the onset of ischemia. Spetzler neurologic functions were assessed post-operatively, and selective perfusion deficits were confirmed by DSA and MRI in all monkeys. Animals undergoing micro-catheter or balloon occlusion demonstrated more profound hemiparesis, larger infarct sizes, lower Spetzler neurologic scores and increased mortality compared to the thrombus occlusion group. In animals injected with the clot, there was no evidence of dissolution, and the thrombus was either near the injection site (M1) or flushed into the superior division of the MCA (M2). All animals survived the M2 occlusion. M1 occlusion with thrombus generated 50% mortality. This study highlighted clinically important differences in these two models, providing a platform for further study of a translational thromboembolic model of acute ischemic stroke.

Ischemic stroke is a leading cause of long-term disability worldwide. A major goal in stroke research is the development of a clinically–relevant translational stroke model. Non-human primates have been suggested as an ideal animal model for preclinical, translational stroke research by the Stroke Therapy Academic Industry Roundtable (STAIR) committee due to translational failures in rodents and significant cerebrovascular, neuroanatomical and biomolecular similarities between nonhuman primates and humans^1^,^2^. Compared to ischemic stroke models achieved by craniotomy in nonhuman primates, ischemia induced by endovascular methods more closely resembles clinical thromboembolic or thrombotic cerebrovascular occlusion. Previous embolic agents have included cyanoacrylate adhesive, silk sutures, polystyrene spheres, nylon thread, microcatheters, balloons and autologous clots^3^,^4^,^5^,^6^,^7^,^8^. However, many of these models poorly mimic clinical thrombus formation, decreasing their clinical utility. In addition, models without an occlusive thrombus preclude the potential of recombinant tissue plasminogen activator (rt-PA) thrombolysis and mechanical recanalization, both of which are currently used in clinical practice.

The advancement of interventional techniques and materials^9^,^10^ has made middle cerebral artery (MCA) access possible, providing a means through which micro-catheter occlusion or thrombus injection can generate an ischemic stroke. Despite previous mechanistic studies, infarct size, survival and neurological function have remained variable among different models. Utilizing micro-catheter occlusion of a distal MCA branch (M1), stroke volume has ranged from 0.1 to 20.5 mL^8^,^11^. Survivals in these ischemic models have also been variable, appearing to be associated with the site of occlusion^12^,^13^. Thrombus dissolution and migration have also been observed following MCA thrombus injection in nonhuman primates^9^. Hemorrhagic transformation and spontaneous reperfusion have been seen in previous studies assessing thrombotic stroke in rhesus monkeys^14^.

This study was designed to analyze and compare two methods of endovascular development of ischemic stroke in adult rhesus monkeys, assessing infarct size, neurological function, survival and MRI characteristics. While these methods have been described in prior studies, there does not yet exist a study built to analyze the variability of key outcome measures between models. Comparing these two endovascular techniques and assessing the variations between them will shed light on developing a robust translational model for acute ischemic stroke in nonhuman primates.

## Results

### Physiological parameters during procedures

**Figure 1:**
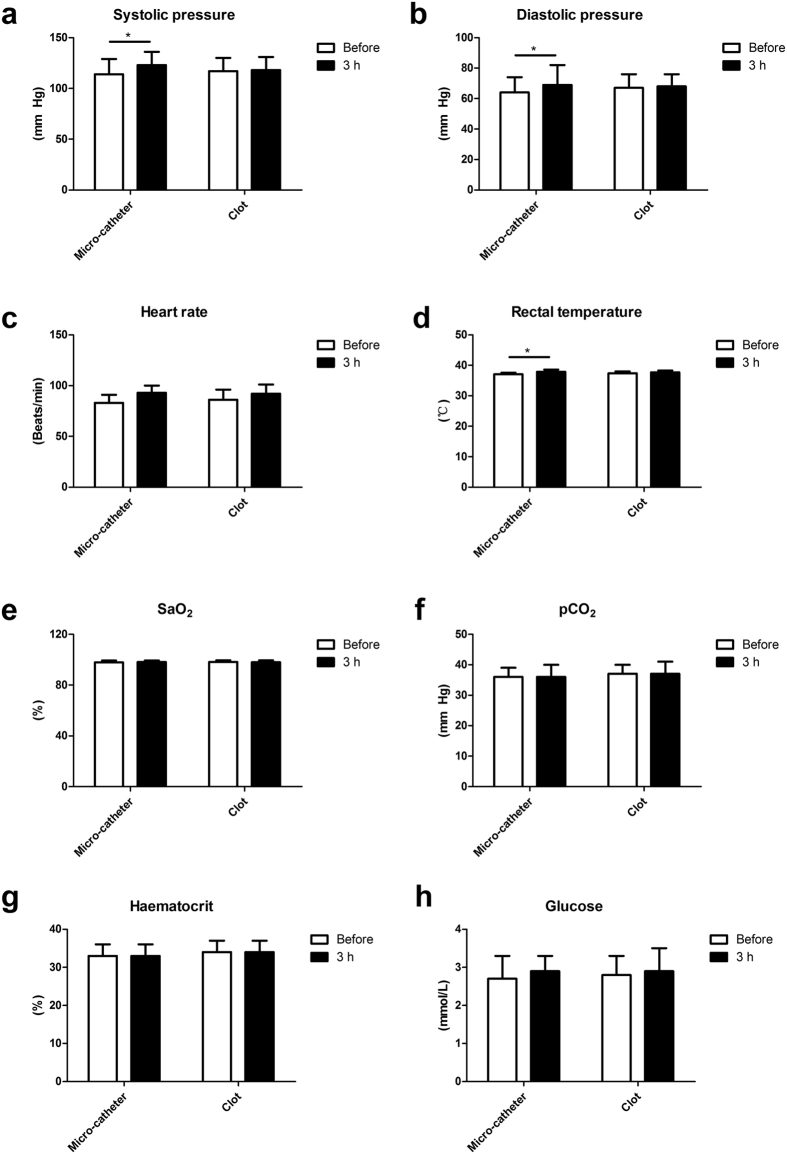
Physiological parameters of animal models subjected to endovascular inducement. (**a**) Systolic blood pressure, (**b**) diastolic blood pressure and (**d**) rectal temperatures were increased at 3 h after the onset of ischemia in the micro-catheter group comparing to baseline values. No significant difference was found for (**c**) heart rate, (**e**) SaO_2_, (**f**) (pCO_2_), (**g**) haematocrit, and (**h**) glucose when adopting the two endovascular methods. *P < 0.05. SaO_2_: oxygen saturation; pCO_2_: partial pressure of carbon dioxide.

The anesthesia and surgical procedures were well tolerated by all monkeys. Systolic blood pressure, diastolic blood pressure and rectal temperatures were increased in the micro-catheter group (Fig. 1) compared to baseline values (P < 0.05), while significant differences were not found for other parameters. There were no significant differences amongst physiological parameters in the thrombus group after embolization (Fig. 1).

### Ischemia in the microcatheter group

**Figure 2:**
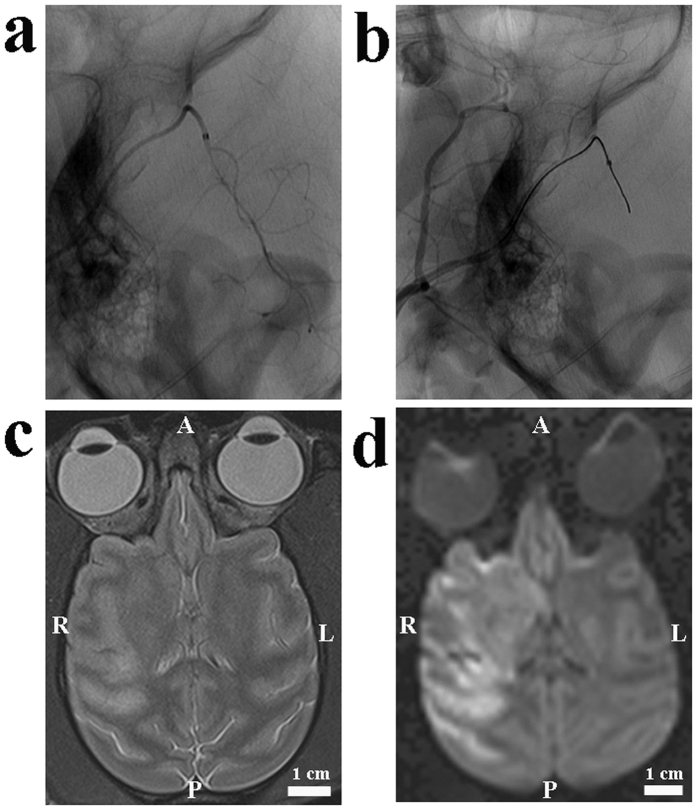
Images from an animal undergoing ischemia through the micro-catheter method. (**a**) A lateral pre-occlusion angiogram demonstrated normal MCA, its divisions and the position of the micro-catheter. (**b**) A lateral post-occlusion angiogram illustrated the perfusion deficit in the M1 main trunk. (**c**) There was a prolonged T2, indicating early vaso-genic edema. (**d**) Diffuse hyper-intensity on DWI scans indicated ischemic lesions. A: anterior; P: posterior; R: right; L: left.

**Figure 3:**
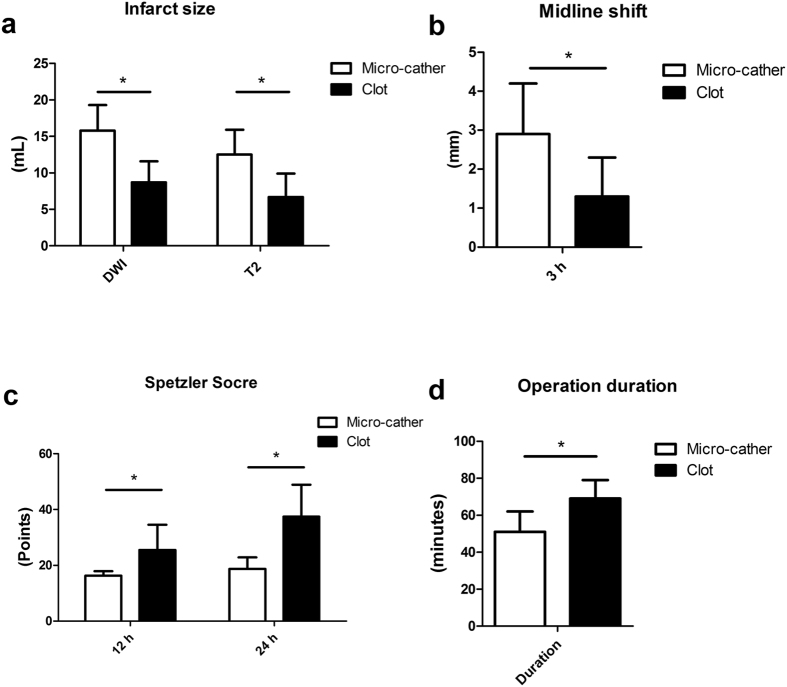
Comparison of infarct size, midline shift, Spetzler Scores and operation duration between the micro-catheter and the thrombus group. (**a**) The micro-catheter group had larger infarct sizes both in DWI and T2 at 3 h following the ischemia. (**b**) The micro-catheter group had an increased midline shift compared to the thrombus group. (**c**) The micro-catheter group had decreased Spetzler scores both at 12 hours and 24 hours when compared to the thrombus group. (**d**) The micro-catheter group had a shorter operation duration compared to the thrombus group. *P < 0.05.

All six rhesus monkeys demonstrated a selective perfusion deficit in the distribution of the MCA based on digital subtraction angiography (DSA). Two animals had obvious collateral flow from the anterior cerebral artery (ACA). Ischemic lesions were clearly seen as focal regions of diffusion restriction on diffusion weighted imaging (DWI) and focal hypointense regions on the apparent diffusion coefficient (ADC) scans, obtained approximately 3 hours after the onset of ischemia. The ischemic regions involved the insular cortex, peri-Sylvian cortex, putamen, caudate nucleus and internal capsule (Fig. 2). The mean ischemic lesion was 15.8 ± 3.5 mL (9.7–20.1 mL) on DWI sequences and 12.5 ± 3.4 mL (6.5–16.2 mL) on T2 sequences, and the mean midline shift was 2.9 ± 1.3 mm (1.2–4.1 mm) (Fig. 3).

All rhesus monkeys had a severe hemiparesis, one was comatose, and the other five were aware of their surroundings but had no response to the examiner. Spetzler neurologic scores were16.3 ± 1.6 (13 or 17) at 12 hours and 18.7 ± 4.1 at 24 hours (17 or 27) following the onset of ischemia. Only one monkey survived the ischemic event and entire post-operative phase (30 days). The animal became more responsive and began self-feeding after 72 hours, with subsequent Spetzler scores of 36 at 7 days and 50 at 30 days. The other five rhesus monkeys were inactive, unable to care for themselves and lacked alertness for a continuous 24-hour period, necessitating sacrifice per protocol.

### Ischemia in the thrombus group

**Figure 4:**
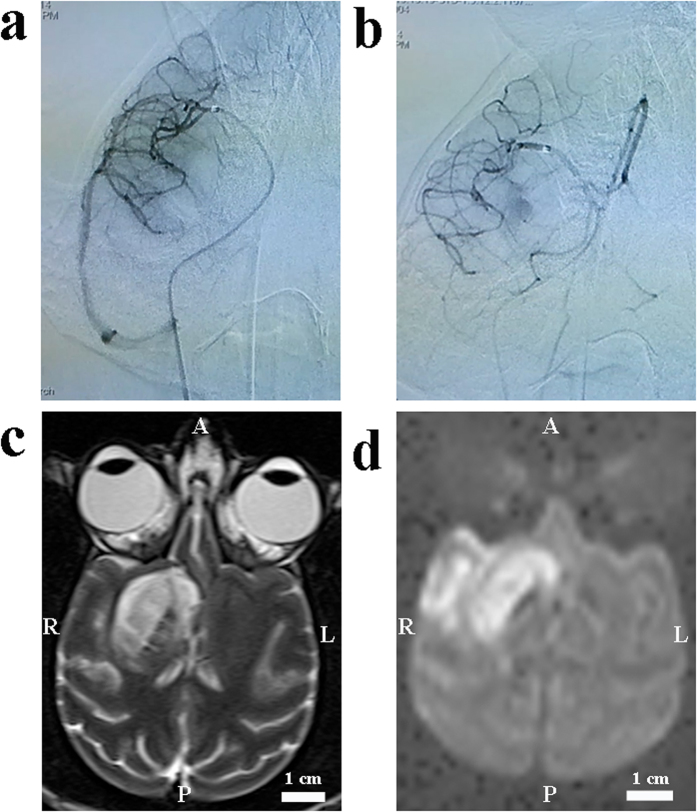
Images from a M1 model of ischemic stroke with an autologous clot. (**a**) A lateral pre-occlusion angiogram demonstrated normal MCA, its divisions and the position of the micro-catheter. (**b**) A lateral post-occlusion angiogram illustrated the perfusion deficit in the M1 divisions. (**c**) Local hyper-intensity was obvious in T2 scans. (**d**) Local hyper-intensity on DWI scans indicated ischemic lesions. A: anterior; P: posterior; R: right; L: left.

**Figure 5:**
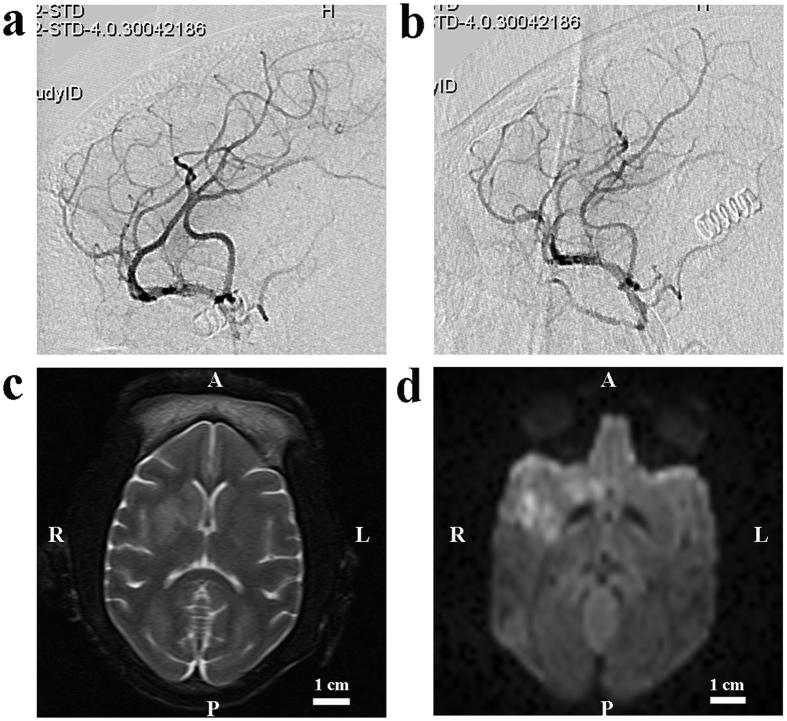
Images from a M2 model of ischemic stroke with an autologous clot. (**a**) A lateral pre-occlusion angiogram demonstrated normal MCA, its divisions and the predominant superior division. (**b**) A lateral post-occlusion angiogram illustrated the perfusion deficit in the superior division. (**c**) There was also an abnormal T2 scans indicating ischemic lesions. (**d**) Local hyper-intensity on DWI scans indicated ischemic stroke. A: anterior; P: posterior; R: right; L: left.

Following clot injection, the thrombus was identified near the injection site (M1) in 4 rhesus monkeys based on DSA imaging (Fig. 4) and had migrated into the superior division of the MCA (M2) in 6 monkeys (Fig. 5). Five monkeys had obvious collateral circulation from the ACA after embolization. The ischemic size in this group was 8.7 ± 2.9 mL (5.5–15.6 mL) on DWI and 6.7 ± 3.2 mL (3.2–13.5 mL) on T2, both of which were lower than those in the micro-catheter group (Fig. 3). The mean midline shift was also lower than that of the micro-catheter group (Fig. 3).

When thrombus remained near M1, the ischemic lesions involved the insular cortex, frontal lobe, putamen and caudate nucleus (Fig. 4). Among animals with stable thrombus at M1, the infarct size was 11.1 ± 3.1 mL (8.3–15.6 mL) on DWI and 9.5 ± 3.1 mL (7.7–13.5 mL) on T2. When the clot migrated into the superior division, the ischemic lesions were mainly located at the insular cortex and frontal lobe (Fig. 5). Infarct size in this group was 7.1 ± 1.3 mL (5.5–9.2 mL) on DWI and 4.9 ± 1.7 mL (3.2–7.1 mL) on T2.

All six rhesus monkeys survived the ischemic events with M2 occlusion. Two animals with M1 occlusion lacked collateral flows and subsequently died on the third day following stroke. The other two monkeys with M1 occlusion had a definitive collateral flow and finally survived. Spetzler neurologic scores were 25.5 ± 9.0 (13–46) at 12 hours and 37.4 ± 11.5 (17–46) at 24 hours following the onset of ischemia, both of which were higher than those in the micro-catheter group (Fig. 3). All surviving animals displayed striking motor and sensory deficits in the left arm, but less so in the left leg.

**Figure 6:**
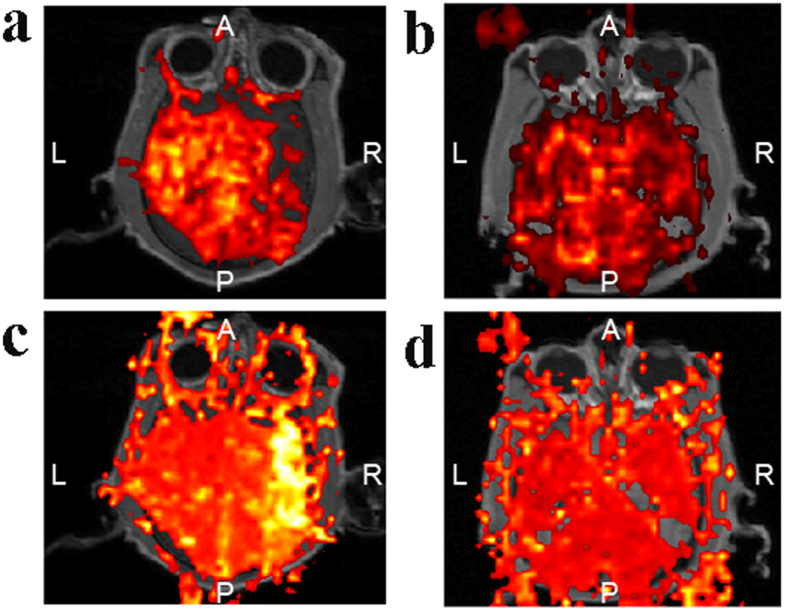
Perfusion deficit in the thrombus group. The perfusion deficit was more obvious based on CBF (**a**) and MTT (**c**) when M1 was blocked, compared with the perfusion deficit according to CBF (**b**) and MTT (**d**) when M2 was occluded. A: anterior; P: posterior; R: right; L: left.

Neither artifact nor hemorrhagic transformation was found on T1 weighted MRI. There was a significant perfusion deficit at 3 hours following the occlusion of M1. Mean transit time (MTT) was prolonged, and cerebral blood flow (CBF) was decreased (Fig. 6). There was also a perfusion deficit at 3 hours after M2 occlusion, although MTT was not significantly prolonged and CBF was only locally decreased.

### Potential factors related to the survival of models

**Figure 7:**
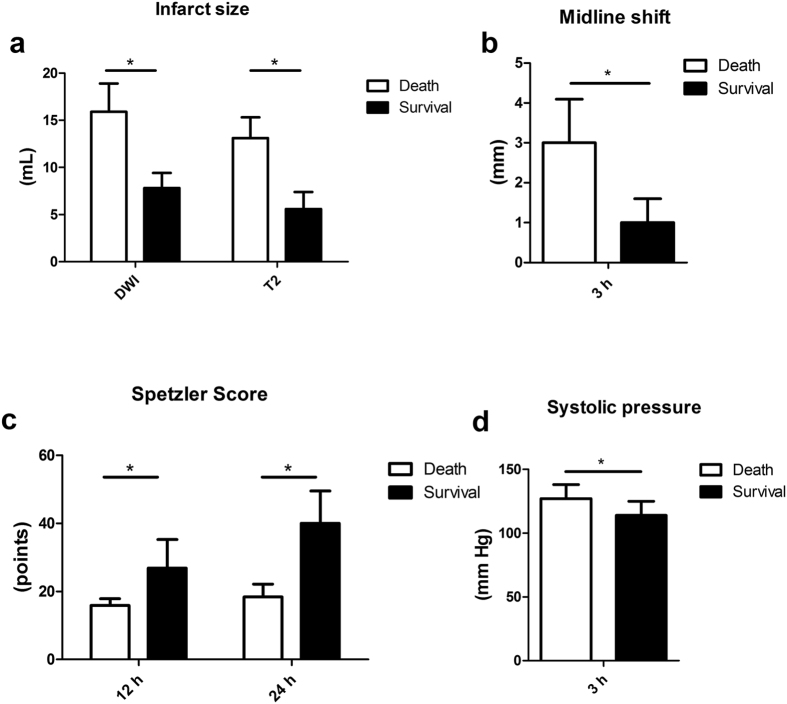
Analysis of possible factors related to the survival of models. (**a**) Dead animals had larger infarct sizes both in DWI and T2 at 3 h following the ischemia. (**b**) Dead monkeys had an increased midline shift compared to alive ones. (**c**) Dead animals had decreased Spetzler scores both at 12 hours and 24 hours when compared to living ones. (**d**) Dead animals had a higher systolic pressure at 3 hours compared to living ones. *P < 0.05.

The animals were secondarily grouped based on the final outcome. Monkeys had the highest survival rate following M2 occlusion with the thrombus injection model (100%, 6/6), then a relatively higher rate (50%, 2/4) following M1 occlusion with the thrombus injection, last following the M1 occlusion with a micro-catheter or balloon (16.7%, 1/6). In addition, surviving animals had a smaller infarct size, a decreased midline shift and a lower systolic blood pressure 3 hours after embolization (Fig. 7). They also had higher post-surgical Spetzler scores at 12 and 24 h after ischemia (Fig. 7).

### Comparison of two methods

Advantages of the micro-catheter method include reproducibility of the site of occlusion, shorter time to ischemia induction, greater control of the ischemia-reperfusion time and a relatively reproducible infarct size (Fig. 3 and Table 1). The advantages of the thrombus injection method include clinically-translatable pathogenesis, relatively small infarct size, greater chance of survival and possibility for thrombolysis treatment. Disadvantages of the balloon and microcatheter include larger infarct size and artifact during MRI, whereas disadvantages of the thrombus injection method include more complicated techniques and decreased control of the exact site of occlusion.

## Discussion

We utilized endovascular techniques to develop two methods of ischemic stroke in rhesus monkeys without craniotomy. In addition to endovascular method, infarct size, site of occlusion, change in systolic pressure and post-surgical Spetzler score at 12 and 24 hours were potentially associated with survival. Most importantly, injecting an enhanced autologous clot was successful and reproducible, holding the potential for further studies of thrombolysis and mechanical recanalization.

The clinical difference between large and small artery occlusions was significant in acute ischemic stroke as it impacted treatment and prognosis^15^. Large artery occlusion generally resulted from the occlusion of the internal carotid artery and proximal MCA with a worse prognosis. Small artery occlusion led to a focal neurologic deficit. The occlusion of M1 in our model, serving as a surrogate for large artery occlusion, resulted in a larger infarct size and higher mortality. The M2 occlusion model led to a more specific occlusion, resulting in a smaller infarct size and increased survival following ischemia. Although control of the exact site of occlusion in the thrombus model was difficult, it created two types of clinically relevant models.

As thrombolysis with rt-PAis the standard of care for stroke within 4.5 hours of symptom onset, many studies have tried to develop animal models through embolus injection or thrombus formation in nonhuman primates^9^,^14^,^15^. One major limitation of thrombus in these models was its fragile nature^9^. Delivery of thrombus by this method under pressure can cause the clot to break down and migrate to the boundary zones of the vascular tree. In another study, spontaneous reperfusion occurred at 2 hours post-injection^14^. We developed a fibrin-rich clot based on experience in rats from a recent study^16^. Red blood cells and platelets were washed out during clot preparation. The improved toughness and flexibility solidified the thrombus and did not break down when injected. There was also no reperfusion based on follow-up MRI analysis.

Anatomical differences may lead to two distinct blockage sites. Human anatomical studies have shown that the middle cerebral artery may exhibit bifurcation, trifurcation or quadrifurcation despite being one of the least variable arteries^17^. Although we did not find variable branches of the MCA in our study, the superior division was predominantly involved. When M2 was occluded, normal blood flow from M1 to the unblocked branch was generally preserved. Our previous study found that rhesus monkeys could survive the ischemic event when the micro-coil was placed at the M2 segment of MCA, while they would die when M1 segment was blocked^10^. So, a survivable primate model could be achieved due to well protected perforator arteries from the main trunk of M1and potential collateral circulations. The death of two animals in the thrombus method may be due to the dramatic hemodynamics changes in local artery following the injection of a clot. When the M1 trunk was blocked with an autologous clot, platelets were prone to aggregate and thrombus continued to grow following the inputted clot due to the disrupted hemostasis^18^. When there were not collateral flows from ACA, these monkeys were prone to have a severe and rapid neurological deterioration, resembling malignant MCA infarction in patients^19^,^20^.

Definite focal perfusion deficit and following neurologic deficit proved the establishment of an ischemic model in rhesus monkeys^9^. Undoubted, it was relatively easy and cost to create an ischemic model in rhesus monkeys with a micro-catheter. However, various studies reported different results. A previous study only reported a relatively smaller infarct size (0.42 mL the smallest) with a Prowler-10 micro-catheter (0.55 mm outside diameter) into a distal branch of M1^11^. It was highly possible that partial perfusion was preserved in that study due to the fact that the original part of the MCA was approximately 0.9–1.0 mm^9^. In other studies, the infarct size ranged from 2.3–29 mL when micro-catheter or balloon was adopted to induce ischemic stroke in cynomolgus monkeys or baboons^12^,^13^,^21^,^22^. Based on previous reports and our experience, it was difficult to use a fixed-size micro-catheter or balloon to block MCA with different size and angel. But, this method had the advantage to evaluate the efficacy and feasibility of local or regional infusion of fibrinolytics, neuro-protective drugs, or ice-cold saline through the micro-catheter in this model before or after the reperfusion^23^,^24^,^25^. Micro-catheter delivery could lead to higher concentrations in target tissue than those achieved with systemic administration^24^. Ischemic models through a micro-catheter or a balloon may be suitable for these efficacy validation studies when a putative neuro-protective agent was considered to be highly effective in rodents and be promised in further clinical trials, such as NXY-059 and PSD-95^26^,^27^,^28^, or for feasibility studies whether local or regional infusions were an adjunctive therapy for neurothrombectomy^25^.

Recent studies found that intra-arterial thrombectomy was a potently effective treatment for acute ischemic patients, especially for those with the occlusion in the distal internal carotid or the proximal middle cerebral artery^28^. Multimodal MRI techniques were imperative for selecting suitable patients for endovascular treatments, other patients for neuro-protectants or collateral augmentation strategies based on the analysis of ischemic core, savable penumbra^25^,^28^,^29^. So, it was an ideal animal model for the analysis of intra-arterial thrombectomy when the M1 trunk was blocked with an autologous clot, especially for analyzing the safety and feasibility of locally or regionally infusing fibrinolytics, neuro-protective drugs, or ice-cold saline following intra-arterial thrombectomy^16^. In addition, intravascular model of stroke in rhesus monkeys could be used to test promising neuroprotective drugs, reperfusion therapies, stem cell implantation, and the effect of rehabilitation on post-stroke recovery^11^. It was also an excellent animal model for the analysis of intra-venous thrombolysis, adjunctive therapies with a long-term survival model. Brain plasticity could be further analyzed at gene, cellular and circuit levels through a rhesus stroke model.

In conclusion, two kinds of ischemic stroke models were developed in rhesus monkeys with a minimally invasive method. Embolization method, infarct site and size, postsurgical Spetzler score had an important influence on survival of ischemic stroke rhesus monkeys.

## Material and Methods

### Animal

A total of 16 adult male rhesus monkeys (Macaca mulatta) were included in this study, aging 8–10 years old, weighting 7.8–10.5 kg. All of them were free of TB, Shigella, Salmonella, Helminths, Ectoparasites, Entamaebahistolytica and B virus. They were individually caged in stainless steel cages in the same room with other animals, and they were fed with commercially prepared monkey food twice daily, plus fruits daily and free water supply. Ischemia was induced in two monkeys with a micro-catheter, in two monkeys with a balloon catheter, in four monkeys with an autologous clot.

All experiments were conducted in compliance with national guidelines and in accordance with the *Guide for the Care and Use of Laboratory Animals*^30^, as sanctioned by the Institute of Laboratory Animal Sciences, Capital Medical University. This study was approved by the Animal Use and Care Board of the Institute of Laboratory Animal Sciences, Capital Medical University.

Criteria for early killing referred to a previous paper^8^, serious neurological or clinical compromise and inability of the animal to care for itself, inactivity and lack of alertness for a continuous 24-hour period.

### Anesthesia

Animals were fasted for at least 12 hours prior to anesthetic induction. Anesthesia was induced with Ketamine (10 mg/kg, IM) and maintained intravenously with propofol (300 μg/kg/min). 18-gauge peripheral venous catheters were placed. Ventilation was controlled (Aridyne 3600; Graham, NC). Intermittent positive pressure ventilation was performed for monkeys with a fixed respiratory rate (22–24 breaths per min), and tidal volume (~120 ml) was adjusted to maintain normal PCO_2_. An indwelling Foley catheter (Baxter) was placed for urinary output monitoring and guidance of fluid management. Non-invasive blood pressure, electrocardiogram, heart rate, oxygen saturation, blood gas and rectal temperature were monitored. When heart rate was lower than 60 bpm, 0.05–0.1 mg of atropine sulfate was given intramuscularly. Rectal temperature was maintained between 37–38 °C.

### Embolization procedure and collateralization assessment

Macaque’s inguinal area was sanitized by iodine and alcohol, and then the area was infiltrated with 2% lidocaine. All animals were given a500 U heparin intravenous bolus, followed by an intravenous infusion of 500 U/h. An18 gage needle was adopted to puncture the femoral artery percutaneously using the Seldinger technique. A sheath was then placed in the femoral artery using a catheter-introducer kit (Radifocus Introducer, Terumo, Tokyo, Japan). A 5-French guiding catheter was introduced over a 0.035-inch guide wire and navigated through the abdominal thoracic aorta, the aortic arch, the brachiocephalic trunk, common carotid artery, and internal carotid artery were catheterized. Papaverine was infused into the guiding catheter continuously and slowly to prevent vasospasm (0.3 mg/min).

In the micro-catheter group, a RebarTM −27 micro-catheter (ev3, Irvine, Calif) with a Traxcess 0.014-inch guiding wire was introduced into the guiding catheter and navigated into the M1 segment of the right MCA in three monkeys. The micro-catheter was placed at the end of M1 segment to induce the ischemic stroke for 3 hours before MRI scanning was performed according to previous reports in rhesus monkeys and baboon^11^,^13^. A balloon catheter (4mm * 10mm, scepter, Terumo, USA) was navigated through the guiding catheter to the end of M1 segment of the right MCA with a 0.014-inch guidewire (Traxcess-14, Terumo, Tokyo) in another three monkeys. The balloon was inflated slowly at the end of M1 segment under the digital C-arm fluoroscopy system until artery occlusion was confirmed by angiograms through the guiding catheter. Ischemic stroke was maintained for 3 hours before MRI scanning was performed. These two methods were generally the same in infarct size, Spetzler scores, prognosis, so they were grouped as the micro-catheter group.

In the thrombus group, blood was drawn from the femoral vein into a polyethylene catheter (Scalp vein set, 25 G), kept at 37 °C for 3 h to allow clot formation, then transferred to keep at 4 °C, one day before the embolization. The method of the clot improvement was according to a recent published paper^16^. A clot (approximately 10 cm in length) was used for embolization. A Rebar^TM^ −18 micro-catheter (ev3, Irvine, Calif) with a Traxcess 0.014-inch guiding wire was introduced into the guiding catheter and navigated into the end of M1 segment of the right MCA. Then the clot was transferred into micro-catheter and flushed into the end of M1 segment with 2 mL saline. Monkeys were transferred to the MRI scanner when ischemia was maintained for 3 hours.

Collateral flow was assessed when the M1 segment of the right MCA was blocked by the micro-catheter or a clot. There was a collateral flow when the blood flow was observed in the occlusion region from the anterior cerebral arteries (ACAs) during DSA examination, based on previous reports in rhesus monkeys and baboons^7^,^31^.

### Post-operation management

After the operation, the interventional devices were withdrawn, the puncture points were compressed for 30 minutes, and then pressure dressed with sterile bandages. Mannitol (1 g/kg, i.v.) and antibiotics (Ceftriaxone Sodium, 50 mg/kg, i.v., daily) was given for those monkeys. In case of pain, an injection of buprenorphine (Buprecare, 20 μg/kg, i.m.) was provided.

### MRI scanning

MRI scanning was performed on a Magnetom Trio MRI Scanner (3.0T; Siemens AG, Siemens Medical Solutions, Erlangen, Germany). Rhesus monkeys were ventilated with an MRI compatible respirator (MRI2550, Surgivet Corp., USA) and monitored with a multi-parameter monitor device (0500-113, Surgivet Corp) under anesthesia. The MRI scans included T1, T2-weighted (high resolution), MR angiograph (MRA), DWI, perfusion weighted imaging (PWI). For perfusion scans, a gadolinium (0.1 mmol/kg) bolus was injected intravenously, starting on the third repetition with a total injection time of 7 s through a peripheral intravenous port. The following protocol was adopted in this study: (1) T2-weighted imaging used fast-spin echo method, TR = 4000 ms, TE = 100 ms, bandwith = 200 Hz/pixel, FOV = 180 mm, slice thickness = 2 mm, 4 averages); (2) DWI, single-shot EPI, TR = 6600 ms, TE = 100 ms, bandwith = 1002 Hz/pixel, FOV = 220 mm, slice thickness = 2 mm, 4 averages; (3) MRA, TR = 20 ms, TE = 3.6 ms, bandwith = 186 Hz/pixel, FOV = 220 mm, slice thickness = 1 mm, 1 average); (4) PWI (single-shot asymmetric spin-echo EPI, TR = 1630 ms, TE = 34 ms, bandwith = 2232 Hz/pixel, FOV = 192 mm, slice thickness = 2 mm, 1 average).

MRI scanning was performed at baseline, and 3 hours following the middle cerebral artery occlusion (MCAO). Stroke volumes were calculated using ITK-Snap contouring software (Pittsburgh) with stacks of average diffusion images reconstructed in three dimensions. Two independent observers analyzed the MRI images in blind.

### Neurological assessments

Neurologic evaluations adopted a commonly used non-human primate scale, the Spetzler neurologic deficit scoring scale, which was fully described in previous rhesus ischemic stroke models^10^,^32^. This scale is weighted heavily on motor function, but also takes into account behavior changes (mental status) and ocular and cranial nerve impairment. Motor function accounts for 70 points, behavior for 20 points, ocular and cranial nerve function for 10 points. Each monkey was assessed with this scale by two independent observers at 12 hours and 24 hours following the MCAO induction.

### Statistical analysis

The means of continuous variables were compared with independent sample t-test, and categorical variables were compared with Fisher exact test. Statistical analysis was performed with SPSS for Windows, version 17.0 (SPSS, Inc.). P < 0.05 was defined as statistical difference.

## Additional Information

**How to cite this article**: Wu, D. *et al.* Endovascular ischemic stroke models of adult rhesus monkeys: a comparison of two endovascular methods. *Sci. Rep.* **6**, 31608; doi: 10.1038/srep31608 (2016).

## Figures and Tables

**Table 1:** Comparison of advantage and disadvantage between two endovascular methods.

	Micro-catheter	Clot
Lodgment of emboli	Easy	Not easy
Reproducibility of blood drop	Yes	Yes
Size of infarct	Large	Modest
MRI compatibility	No	Yes
Potential rt-PA treatment	No	Yes
